# Modular assembly of synthetic proteins that span the plasma membrane in mammalian cells

**DOI:** 10.1186/s12896-016-0320-7

**Published:** 2016-12-09

**Authors:** Anam Qudrat, Kevin Truong

**Affiliations:** 1 Institute of Biomaterials and Biomedical Engineering, University of Toronto, 164 College Street Room 407, Rosebrugh Building, Toronto, ON M5S 3G9 Canada; 2 Edward S. Rogers, Sr. Department of Electrical and Computer Engineering, University of Toronto, 10 King’s College Circle, Toronto, ON M5S 3G4 Canada

**Keywords:** Transmembrane proteins, Modular assembly, Endoplasmic reticulum, Plasma membrane, Protein engineering, Synthetic biology

## Abstract

**Background:**

To achieve synthetic control over how a cell responds to other cells or the extracellular environment, it is important to reliably engineer proteins that can traffic and span the plasma membrane. Using a modular approach to assemble proteins, we identified the minimum necessary components required to engineer such membrane-spanning proteins with predictable orientation in mammalian cells.

**Results:**

While a transmembrane domain (TM) fused to the N-terminus of a protein is sufficient to traffic it to the endoplasmic reticulum (ER), an additional signal peptidase cleavage site downstream of this TM enhanced sorting out of the ER. Next, a second TM in the synthetic protein helped anchor and accumulate the membrane-spanning protein on the plasma membrane. The orientation of the components of the synthetic protein were determined through measuring intracellular Ca^2+^ signaling using the R-GECO biosensor and through measuring extracellular quenching of yellow fluorescent protein variants by saturating acidic and salt conditions.

**Conclusions:**

This work forms the basis of engineering novel proteins that span the plasma membrane to potentially control intracellular responses to extracellular conditions.

**Electronic supplementary material:**

The online version of this article (doi:10.1186/s12896-016-0320-7) contains supplementary material, which is available to authorized users.

## Background

The ability to reliably engineer a protein that spans the plasma membrane (PM) allows synthetic control over the communication of the cell with its extracellular environment [1]. The intercellular and extracellular environment is separated by the PM, where proteins spanning the PM facilitate diverse cellular processes such as signal transduction, cell-cell contact and molecular transport [2]. Many proteins spanning the PM contain an N-terminal signal peptide that is recognized by the signal recognition particle (SRP) and recruited to the ribosomes on the endoplasmic reticulum (ER) for translation [3, 4]. Subsequently the signal peptide is cleaved off by the signal peptidase. Signal peptides are usually 20 to 60 amino acids consisting of initially, a hydrophilic segment followed by a hydrophobic segment and finally a signal peptidase cleavage site [5, 6]. Proteins spanning the PM require at least one transmembrane domain (TM) consisting of mostly hydrophobic residues, that at first, anchors the protein to the ER membrane, among other retention signals. Next, the PM-bound proteins are packaged into coated vesicles (i.e. COP II vesicles) and shuttled from the ER to the Golgi apparatus for further processing [7]. Upon exiting the Golgi apparatus, they are again packaged into vesicles (i.e. clathrin-coated vesicles) and shuttled to the PM [7, 8]. After the membrane of the vesicle fuses with the PM, the membrane-spanning protein is delivered to the PM.

While many groups have created membrane-tethered proteins for use as research tools and therapeutics [9, 10], it is unclear what the minimum necessary components to engineer a protein that spans the PM are. Examples of membrane-tethered proteins from other groups include antibody fragments comprising pseudo-receptors to activate the immune system [11], secreted cytokines to induce specific antitumor immune responses with reduced systemic toxicity [12, 13] and enzymes to activate prodrugs [14]. To enhance cell surface expression of membrane-tethered proteins, design strategies have focused on the careful selection of the TM, the cytoplasmic tail and the extracellular juxtamembrane linker [15]. Elements such as the transmembrane domain and the cytoplasmic tail of the murine B7-1 antigen and the hinge-CH_2_-CH_3_ region of the human immunoglobulin G (IgG_1_) heavy chain have been established to enhance protein expression on the cell surface [16, 17]. While the minimum sequences required to target specific proteins such as the respiratory syncytial virus F protein [18] and the SNARE protein SNAP-25 [19] have been elucidated in independent studies previously, these specific minimum sequences could differ between target proteins.

To determine the minimum necessary components to engineer a protein that spans the PM with a defined orientation, we used a modular synthetic biology approach to assemble the protein. Previously, our group employed a similar approach to determine that the minimum component to engineer a protein that spans the ER is a single TM at an arbitrary location in the protein [20]. An N-terminal signal peptide was not necessary to deliver the protein to the ER [20]. Here, we confirm that while an N-terminal TM directs the protein for translation in the ER, the engineered protein is still strongly retained in the ER. The addition of a downstream peptidase cleavage site to this TM further enhances the exit of the engineered protein from the ER, thereby decreasing its retention in the ER. Finally, a second TM strongly increases retention of the engineered protein in the PM. Furthermore, the orientation (i.e., extracellular vs. intracellular) of the engineered proteins were determined by fluorescence quenching and detection of intracellular Ca^2+^ signaling.

## Results and discussion

### A signal peptidase cleavage site enhances sorting out of the ER

**Fig. 1:**
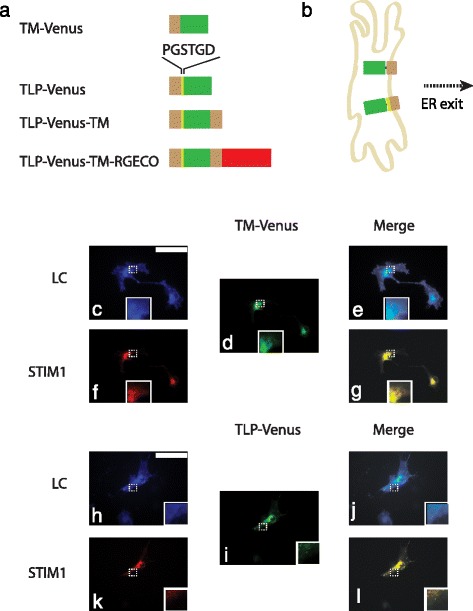
A signal peptidase cleavage site enhances sorting out of the ER. Schematic layout of fusion proteins constructed in the study (**a**). Pictorial representation of receptors exiting the ER (**b**). COS-7 cells transfected with the plasma membrane labelled Lyn-Ceru showed a matte-like appearance (**c**) while those transfected with the ER labelled STIM1-mRFP showed a web-like fluorescence distribution (f inset). TM-Venus showed a web-like fluorescence distribution similar to STIM1-mRFP (**d** and **f**). Merged images illustrate resultant co-localization (**e** and **g**). COS-7 cells transfected with TLP-Venus (containing an additional signal peptidase cleavage site) had a less web-like fluorescence distribution when compared to STIM1-mRFP (**h**, **i** and **k**). Merged images illustrate resultant co-localization (**j** and **l**). Amino acid single letter codes PGSTGD represent proline, glycine, serine, threonine, glycine and aspartic acid, respectively. TM: transmembrane domain TLR4, TLP: fusion of TM with signal peptidase cleavage site from human immunoglobulin K, RGECO: red fluorescent genetically encoded Ca^2+^ indicator, ER: endoplasmic reticulum, LC: Lyn-Ceru, STIM1: stromal interaction molecule 1. Scale bars are 10 μm. Images are false colored: CFP, cyan; YFP, green; mRFP, red. All insets show zoomed regions (4x) of structures in dotted rectangles. All experiments were repeated at least 3 times

While an N-terminal TM traffics the protein to the ER, an additional signal peptidase cleavage site downstream of this TM enhanced sorting out of the ER. The protein named TM-Venus was created as the tandem fusion of the TM from the human toll-like receptor 4 (^632^TIIGVSVLSVLVVSVVAVLVY^652^) (TM) and the yellow fluorescent protein (YFP) mutant Venus [21] (Fig. 1a,b). Although the TM domain from TLR4 was used in this study, TM domains from other proteins should behave similarly because in our previous work [20], the TM domain from platelet-derived growth factor receptor behaved indistinguishably to TLR4. To label the plasma membrane, the protein Lyn-Ceru was used consisting of the tandem fusion of N-terminal localization sequence of Lyn kinase (^1^MGCIKSKGKDSA^12^) and the cyan fluorescent protein variant Cerulean [22]. Following post-translational modification (i.e. palmitoylation of the cysteine amino acid), Lyn-Ceru localizes to the cytoplasmic side of the plasma membrane. Likewise, to label the ER, the protein STIM1-mRFP was used, consisting of the tandem fusion of the stromal interaction molecule 1 (STIM1, an endogenous ER-Ca^2+^ sensor labeling the ER membrane [23]) and the monomeric red fluorescent protein (mRFP) [24]. Mammalian cells were co-transfected with TM-Venus and Lyn-Ceru to compare localization with the PM or TM-Venus and STIM1-mRFP to compare localization with the ER (Fig. 1c-g). TM-Venus showed a web-like fluorescence distribution similar to STIM1-mRFP as would be expected from ER localization (Pearson’s correlation coefficient (PCC) = 0.87 ± 0.01) (Table 1, Fig. 1f-g) (*n* = 3 experiments, with at least 9 cells per experiment). In contrast, the TM-Venus had a starkly different fluorescence distribution compared to Lyn-Ceru that labels the plasma membrane (Table 1, PCC = 0.75 ± 0.04). There was a significant difference between ER and plasma membrane localization (*p* < 0.01). Notably, Lyn-Ceru labels the PM distinctly depending on cell spreading. Cells that spread well (e.g. COS-7 cells) show a matte-like fluorescence appearance, whereas cells that do not spread as well (e.g. CHO cells (Additional file 1: Figure S1)) often exhibit a fluorescence that outlines the boundary of the cell (Fig. 1c-e). To enhance sorting out of the ER, the protein named TLP-Venus was created as the tandem fusion of TM, the signal peptidase cleavage site from human immunoglobulin K (^16^PGSTGD^21^) and Venus. Cells were co-transfected with TLP-Venus and either Lyn-Ceru or STIM1-mRFP. TLP-Venus had a less web-like fluorescence distribution when compared to STIM1-mRFP (PCC = 0.80 ± 0.10) (Table 1, Fig. 1h-l) (*n* = 3 experiments, with at least 9 cells per experiment) and while it did not look plasma membrane when measured against Lyn-Ceru (Table 1, PCC = 0.83 ± 0.05) either, it improved sorting compared to the TM-Venus case (Fig. 1d,i) (*n* = 3 experiments, with at least 9 cells per experiment). Repeated trials in another mammalian cell line (i.e. CHO cells) yielded similar results (Additional file 1: Figure S1 a-j) (*n* = 3 experiments, with at least 9 cells per experiment). Thus, the signal peptidase cleavage site enhanced sorting out of the ER.

**Table 1:** Ratios of Pearson’s correlation coefficient (PCC) of fusion proteins in COS-7 cells

Fusion protein	Marker		PCC^a^	Ratio^b^
	LC	STIM1		
TM-Venus	x		0.75 ± 0.04	0.57
		x	0.87 ± 0.01	
TLP-Venus	x		0.83 ± 0.05	0.71
		x	0.80 ± 0.10	
TLP-V-TM	x		0.96 ± 0.001	0.93
		x	0.64 ± 0.10	

PCCs of fusion proteins with markers LC (Lyn-Ceru) and STIM1 localized to the PM and ER, respectively

^a^Average PCCs calculated from 3 independent experiments with at least 3 cells per experiment

^b^Ratio of PM accumulation calculated as: $=\kern0.5em \frac{Ratio\left(LC: fusion\kern0.5em  protein\right)- Ratio\left(LC: STIM1\right)}{Ratio\left(LC:LC\right)- Ratio\left(LC: STIM1\right)},$ where Ratio (LC:STIM1) = 0.42 and Ratio (LC:LC) = 1

### A second TM anchors the protein on the PM

**Fig. 2:**
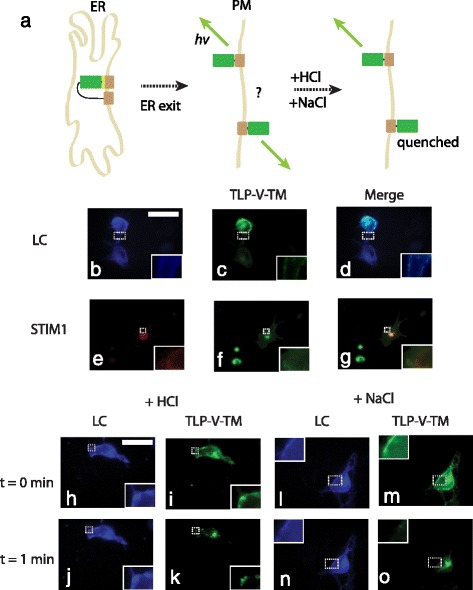
An additional transmembrane domain anchors proteins on the PM with the fluorescent protein, Venus, facing extracellularly. Pictorial representation of receptor anchoring and orientation (**a**). COS-7 cells transfected with the plasma membrane labelled Lyn-Ceru showed a matte-like appearance (**b** and **c**) while those transfected with the ER labelled STIM1-mRFP showed a web-like fluorescence distribution (**e** and **f**). Merged images illustrate resultant co-localization (**d** and **g**). TLP-V-TM skewed more towards a matte-like plasma membrane appearance (**b** and **c**). Fluorescence quenching with [30 μM]_f_ HCl showed no effect on membrane-labelled Lyn-Ceru at time = 0 and time = 1 min (**h** and **i**) but did sequester Venus (**j** and **k**). Likewise, fluorescence quenching with [60 μM]_f_ NaCl showed no effect on membrane-labelled Lyn-Ceru at time = 0 and time = 1 min (**l** and **m**) but again sequestered Venus (**n** and **o**). ER: endoplasmic reticulum, PM: plasma membrane, *hv*: emitted fluorescent light, HCl: hydrogen chloride, NaCl: sodium chloride, TM: transmembrane domain TLR4, TLP: fusion of TM with signal peptidase cleavage site from human immunoglobulin K, V: Venus fluorescent protein, LC: Lyn-Ceru, STIM1: stromal interaction molecule 1. Scale bars are 10 μm. Images are false colored: CFP, cyan; YFP, green; mRFP, red. All insets show zoomed regions (4x) of structures in dotted rectangles. All experiments were repeated at least 3 times

While TLP-Venus enhances sorting out of the ER, a second TM helps anchor and accumulate proteins on the PM (Fig. 2a). To anchor proteins on the plasma membrane, the construct TLP-Venus-TM was created. Proteins sorted from the ER are directed via COP II vesicles to the trans Golgi network (TGN) for further processing before being packaged into recycling endosomes (RE) or post-Golgi transport intermediates (PGTIs) for delivery to the plasma membrane [3, 7, 25]. From the TGN, proteins are committed to one of two secretory pathways: *constitutive* secretion involves the continuous release of proteins from the cell whereas *regulated* secretion retains proteins intracellularly before an external signal initiates release [3]. Ultimately, the secretory vesicle fuses with the PM and the protein is either to be secreted or retained on the plasma membrane [26, 27]. It is plausible that the presence of an anchoring domain (e.g. TM) on the protein will enhance retention of the protein on the PM. In theory, since the TLP contains a signal peptidase cleavage site, the first TM within TLP cannot serve as permanent anchoring domain, however the second TM can serve in that function. When TLP-Venus-TM was co-transfected with either Lyn-Ceru (PCC = 0.96 ± 0.001) or STIM1-mRFP (PCC = 0.64 ± 0.10) (Table 1) in COS-7 cells, the protein distribution skewed more towards the plasma membrane as expected (Fig. 2b-g) (*n* = 3 experiments, with at least 9 cells per experiment). The matte-like fluorescence was significantly similar to that of Lyn-Ceru unlike the web-like detail seen in STIM1-mRFP (*p* < 0.01, Fig. 2c,f) (*n* = 3 experiments, with at least 9 cells per experiment). Repeated trials in another mammalian cell line (i.e. CHO) yielded similar results (Additional file 1: Figure S1 k-o) (*n* = 3 experiments, with at least 9 cells per experiment). To estimate the relative degree of PM accumulation between TM-Venus, TLP-Venus and TLP-Venus-TM, we compared their co-localization to STIM1-mRFP and Lyn-Ceru in the same cell (Table 1). If the co-localization is the same as STIM1-mRFP, the ratio of PM accumulation is 0; if similar to Lyn-Ceru, 1. The ratio of PM accumulation for TM-Venus, TLP-Venus and TLP-Venus-TM was calculated as 0.57, 0.71, and 0.93, respectively, suggesting each modification increased accumulation to the PM.

To compare the rate of trafficking of TM-Venus, TLP-Venus and STIM1-mRFP to the PM, we performed a cyclohexamide chase assay in CHO cells. Since cyclohexamide inhibits protein synthesis, we can determine protein accumulation on the PM over time by observing the co-localization with Lyn-Ceru. At time 0 h, both TM-Venus and STIM1-mRFP had lower and similar PCC with Lyn-Ceru (PCC = ~0.43 ± 0.20) whereas TLP-Venus had a higher and statistically different PCC with Lyn-Ceru (PCC = 0.69 ± 0.13) (Additional file 2: Figure S2). At time 1, 4 and 12 h, cyclohexamide enhanced accumulation of both TM-Venus, TLP-Venus to the PM at a similar rate, but as expected did not enhance accumulation of STIM1-mRFP to the PM (Additional file 2: Figure S2). It is worth noting that in our previous study [20], we created proteins of the TM-fluorescent protein-TM architecture where the fluorescence was mostly ER, so the signal cleavage site is needed for enhanced exiting of the ER.

### Venus faces the extracellular side in the TLP-Venus-TM construct

To determine the orientation of TLP-Venus-TM, fluorescence quenching was performed using saturating acidic and salt conditions. Under saturated acidic conditions, rapid (<1 ms) protonation of the protein backbone physically expands and thus denatures the barrel-like domain housing fluorescence-producing chromophore phenols. This makes the chromophores also susceptible to protonation leading to a decrease in fluorescence [28, 29]. Fluorescence quenching is also seen with an increased salt concentration which reduces the repulsion between surface charges on the protein causing it to collapse via van der Waals forces [30]. Since transport through ion channels is often needed to influx these stimuli (i.e. H^+^, Na^+^, Cl^−^), they do not freely permeate the hydrophobic PM bilayer due to charge constraints. Within a minute of treatment with either [30 μM]_f_ of HCl or [60 μM]_f_ of NaCl, a change in the expected fluorescence distribution of TLP-Venus- TM was observed (Fig. 2h-o) (*n* = 9) (*n* = 3 experiments, with at least 9 cells per experiment). Noticeably, only the plasma membrane localized protein (i.e. Venus facing extracellularly) was quenched as it was accessible to the quenching agents while those proteins being processed intracellularly remained intact. At time = 0, the fluorescence appears strongly matte-like while within a minute post treatment, this trait vanishes and a previously masked fluorescence of the intracellular proteins being processed becomes clearly visible. Since the fluorescence distribution of Lyn-Ceru which is facing the cytoplasmic side of the PM remains identical pre- and post-treatment, Venus in the TLP-Venus-TM construct should be extracellular (Fig. 2h, j, l, n) (*n* = 3 experiments, with at least 9 cells per experiment).

### An ancillary fragment, R-GECO faces the cytoplasmic side in the TLP-Venus-TM-R-GECO

**Fig. 3:**
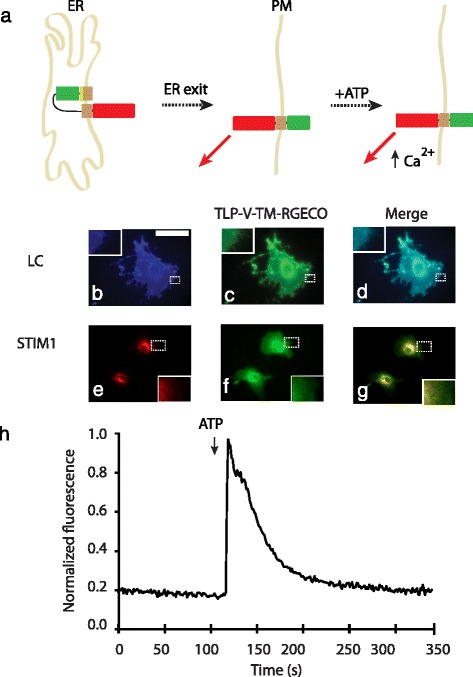
An ancillary fragment, R-GECO, is cytoplasmic and responsive to an induced Ca^2+^ influx. Pictorial representation of receptor orientation and response (**a**). COS-7 cells transfected with the plasma membrane labelled Lyn-Ceru showed a matte-like appearance (**b** and **c**) while those transfected with the ER labelled STIM1-mRFP showed a web-like fluorescence distribution (**e** and **f**). TLP-V-TM-RGECO showed a matte-like fluorescence distribution similar to Lyn-Ceru (**b** and **c**). Merged images illustrate resultant co-localization (**d** and **g**). Upon stimulation with 10 μM ATP, the Ca^2+^ concentration rapidly increased to a peak within a few minutes and then gradually declined to its basal level within two to three minutes (**h**). ER: endoplasmic reticulum, PM: plasma membrane, ATP: adenosine triphosphate, TM: transmembrane domain TLR4, TLP: fusion of TM with signal peptidase cleavage site from human immunoglobulin K, V: Venus fluorescent protein, RGECO: red fluorescent genetically encoded Ca^2+^ indicator, LC: Lyn-Ceru, STIM1: stromal interaction molecule 1. Scale bars are 10 μm. Images are false colored: CFP, cyan; YFP, green; mRFP, red. All insets show zoomed regions (4x) of structures in dotted rectangles. All experiments were repeated at least 3 times

A protein domain fused C-terminal to TLP-Venus-TM faces the cytoplasm. To determine the orientation of this domain, the construct TLP-Venus-TM-R-GECO was created, where R-GECO is a red fluorescent genetically encoded Ca^2+^ indicator [27] (Fig. 3a). The extracellular Ca^2+^ concentration in the media is approximately 2 mM while the intracellular concentration ranges from 0.1 to 1 μM - a difference of a thousand times. R-GECO being sensitive to the smaller range would not be responsive to any intracellular Ca^2+^ transient if it were facing extracellularly [28]. Co-transfection of TLP-Venus-TM-RGECO and Lyn-Ceru in mammalian cells confirmed its localization to the plasma membrane as indicated by the cell’s matte-like appearance (PCC = 0.83 ± 0.09) (Fig. 3b-g) (*n* = 3 experiments, with at least 9 cells per experiment). The red fluorescence from R-GECO was initially weak but increased in intensity in response to a Ca^2+^ transient. Upon stimulation with 10 μM ATP, the Ca^2+^ concentration rapidly increased to a peak within a few seconds and then gradually declined to its basal level within two to three minutes (Fig. 3h) (*n* = 3 experiments, with at least 9 cells per experiment). It has been established that ATP activates endogenous P2Y receptors in cells to stimulate IP_3_ production and subsequent Ca^2+^ release from the endoplasmic reticulum [29]. The measured Ca^2+^ response shows that the domain following the TM was intracellular.

## Conclusions

Using a modular synthetic biology approach to assemble proteins, we verify that proteins are targeted to the ER membrane with the inclusion of an N-terminal TM. With the addition of a peptidase cleavage site downstream of the TM, the protein has enhanced exit from the ER; with the further addition of a second TM, the protein can efficiently anchor and accumulate on the PM. Furthermore, fluorescence quenching and intracellular Ca^2+^ responses were used to distinguish domain orientation. This study establishes a foundation to engineer novel synthetic proteins that span the PM that can be used to potentially direct intracellular signaling in response to changing extracellular conditions. For instance, extracellular domains could be selected to dimerize in response to a synthetic molecule, which in turn cause specific intracellular kinase domains to autophosphorylate and activate desired downstream signaling cascades.

## Methods

### Plasmids

STIM1-mRFP, Lyn-Ceru and TM_TLR4_ were subcloned previously [20, 31]. TLP was created by fusing the transmembrane domain (TM_TLR4_) with signal peptidase cleavage site from human immunoglobulin K (^16^PGSTGD^21^) through overlap PCR and insertion into the pCfvtx3 [31, 32]. All subsequent fusion proteins with the yellow fluorescent protein (YFP) mutant Venus and the monomeric red fluorescent protein (mRFP) were subcloned as previously described [32–34]. The fragment R-GECO was amplified from CMV-NLS-R-GECO, which was a gift from Robert Campbell (Addgene plasmid #32462) and inserted into our cassette subcloning methodology. All plasmids were transformed in *Escherichia coli* and were isolated using the Mini-prep kit (Invitrogen).

### Cell culture and transfection

COS-7 and CHO cells were maintained in Dulbecco’s Modified Eagle’s Medium containing 25 mM D-glucose, 1 mM sodium pyruvate and 4 mM L-glutamine (Invitrogen, Carlsbad, CA) with 10% supplemented Fetal Bovine Serum (FBS) (Sigma Aldrich, St. Lois, MO) in T25 flasks (37 °C and 5% CO_2_). Cells were passaged at 95% confluency using 0.05% TrypLE with Phenol Red (Invitrogen) and seeded onto 24-well Multiwell Plates (Falcon, Corning, NY) at a dilution of 1:20. Cells were transiently transfected using Lipofectamine 2000 according to manufacturer’s protocols (Invitrogen). Post-transfection, cells were treated with 0.05% TrypLE with Phenol Red (Invitrogen) and plated in 96-well tissue culture plates (Sarstedt, Numbrecht, Germany) at a dilution of 1:4 for imaging.

### Quantitative cyclohexamide chase assay

Twenty-four hours post-transfection, CHO cells were incubated with 10 μg/mL of cyclohexamide (Sigma Aldrich, St. Lois, MO) and imaged after 1, 4 and 12 h. Results were quantified with one-tailed two-sample Student’s t-test between the time points and significance was reported.

### Illumination and imaging

Imaging was performed using an inverted IX81 microscope with Lambda DG4 xenon lamp source and Tuscen H674-ICE CCD camera with a 40x objective (Olympus). Filter excitation (EX) and emission (EM) bandpass specifications were as follows (in nm): CFP (EX: 438/24, EM: 482/32), YFP (EX: 500/24, EM: 542/27), RFP (EX: 580/20, EM: 630/60) (Semrock). Image acquisition and analysis was done with ImageJ and μManager software [35, 36].

### Co-localization analysis

Colocalization coefficients such as Pearson’s correlation coefficient (PCC) singly and in conjunction with Costes’ thresholding and Van Steensel’s cross correlation coefficients (CCF) analysis were calculated using the JaCoP plugin for ImageJ [37]. Only PCCs were cited as there were no significant differences between the various calculation methods.

### Statistical analysis

For all experiments, mean PCC ± standard deviation is cited. Let the combination of plasmids be termed as follows: TM-V + LC = A, TM-V + STIM1 = B, TLP-V + LC = C, TLP-V + STIM1 = D, TLP-V- TM + LC = E, TLP-V- TM + STIM1 = F, TLP-V-TM-RGECO + LC = G, TLP-V-TM-RGECO + STIM1 = H. A two-tailed two-sample Student’s t-test was performed under the following null hypotheses: H_0_: μ_A_ = μ_B_, μ_C_ = μ_D_, μ_E_ = μ_F_, μ_G_ = μ_H_, μ_E_ = μ_G_ and μ_F_ = μ_H_. A one-tailed two-sample t test was performed under the following alternative hypotheses: H_A_: μ_A_ < μ_C_, μ_B_ > μ_D_, μ_E_ > μ_A_, μ_E_ > μ_C_, μ_F_ < μ_B_, μ_F_ < μ_D_, μ_G_ > μ_A_, μ_G_ > μ_C_, μ_H_ < μ_B_ and μ_H_ < μ_D_. Only significant results were cited as p = significance.

## Additional files

Additional file 1: Figure S1. Fluorescence images of CHO cells transfected with the plasma membrane labelled Lyn-Ceru and ER labelled STIM1-mRFP markers. CHO cells transfected with the plasma membrane labelled Lyn-Ceru showed a matte-like appearance (a, f and k) while those transfected with the ER labelled STIM1-mRFP showed a web-like fluorescence distribution (d, i and n). TM-Venus (b), TLP-Venus (g) or TLP-V-TM (l) peptides show a shift in fluorescence distribution from a wholly ER (b) to a slight (g) and then an entirely membrane appearance (l). Merged images illustrate resultant co-localization (c, e, h, j, m and o). TM: transmembrane domain TLR4, TLP: fusion of TM with signal peptidase cleavage site from human immunoglobulin K, V: Venus fluorescent protein, LC: Lyn-Ceru, STIM1: stromal interaction molecule 1. Scale bars are 10 μm. Images are false colored: CFP, cyan; YFP, green; mRFP, red. All insets show zoomed regions (4x) of structures in dotted rectangles. All experiments were repeated at least 3 times. (PDF 9198 kb)
Additional file 2: Figure S2. Line graph showing changes in distribution of plasma membrane labelled Lyn-Ceru compared with ER-labelled STIM1-mRFP, TM-Venus and TLP-Venus in CHO cells, post cyclohexamide treatment. CHO cells transfected with the plasma membrane labelled Lyn-Ceru and the ER labelled STIM1-mRFP, TM-Venus or TLP-Venus. Cells were imaged initially and after 1, 4 and 12 h after incubation with 10 μg/mL cyclohexamide. Y-axis shows PCC of the STIM1-mRFP, TM-Venus or TLP-Venus with Lyn-Ceru. TM: transmembrane domain TLR4, TLP: fusion of TM with signal peptidase cleavage site from human immunoglobulin K, V: Venus fluorescent protein, LC: Lyn-Ceru, STIM1: stromal interaction molecule 1. Error bars indicate s.d. Star indicates significance of *p* < 0.05. (PDF 238 kb)

## Abbreviations

ATP: Adenosine triphosphate
CCF: Van Steensel’s cross correlation coefficients
ER: Endoplasmic reticulum
IgG: Immunoglobulin G
IP_3_: Inositol triphosphate
mRFP: Monomeric red fluorescent protein
PCC: Pearson’s correlation coefficient
PGTIs: Post-Golgi transport intermediates
PM: Plasma membrane
RE: Recycling endosomes
R-GECO: Red fluorescent genetically encoded Ca^2+^ indicator
SRP: Signal recognition particle
STIM1: Stromal interaction molecule 1
TGN: Trans Golgi network
TM: Transmembrane domain
YFP: Yellow fluorescent protein
