# Supplementary figures and images for: Modular assembly of synthetic proteins that span the plasma membrane in mammalian cells
Source: BMC Biotechnol. 2016 Dec 9;16:88. doi: 10.1186/s12896-016-0320-7 (PMC5148844; doi:10.1186/s12896-016-0320-7)

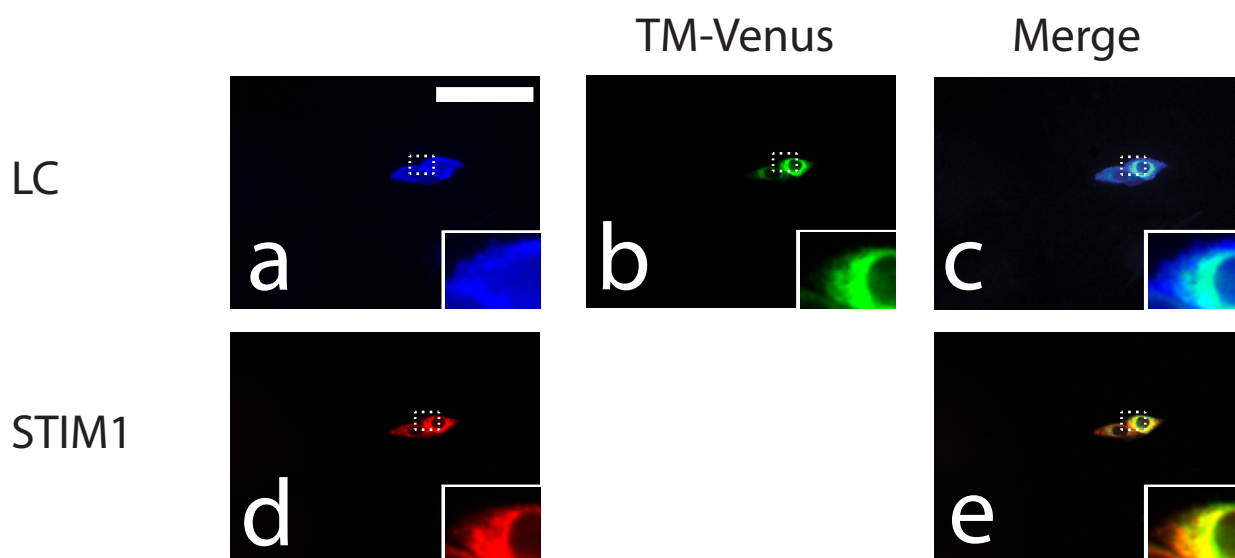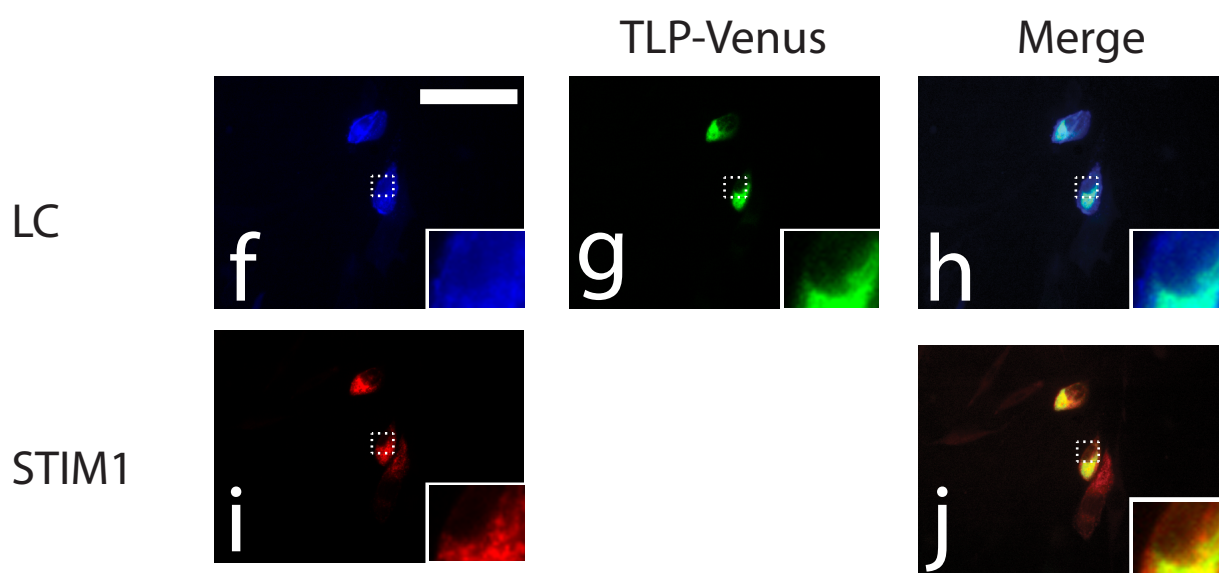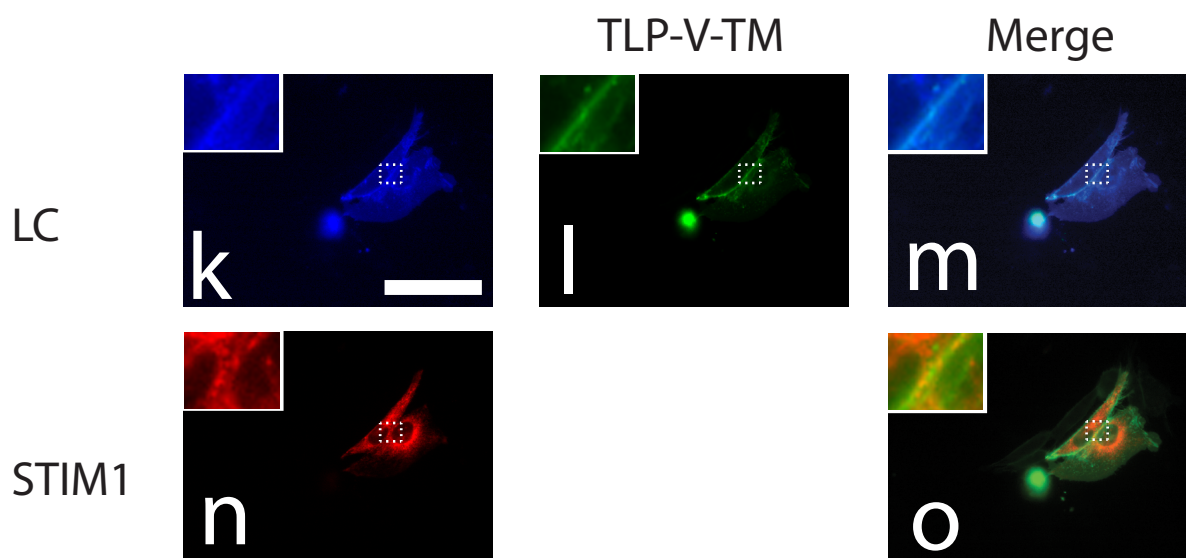

Supplement: Additional file 1: Figure S1. — Fluorescence images of CHO cells transfected with the plasma membrane labelled Lyn-Ceru and ER labelled STIM1-mRFP markers. CHO cells transfected with the plasma membrane labelled Lyn-Ceru showed a matte-like appearance (a, f and k) while those transfected with the ER labelled STIM1-mRFP showed a web-like fluorescence distribution (d, i and n). TM-Venus (b), TLP-Venus (g) or TLP-V-TM (l) peptides show a shift in fluorescence distribution from a wholly ER (b) to a slight (g) and then an entirely membrane appearance (l). Merged images illustrate resultant co-localization (c, e, h, j, m and o). TM: transmembrane domain TLR4, TLP: fusion of TM with signal peptidase cleavage site from human immunoglobulin K, V: Venus fluorescent protein, LC: Lyn-Ceru, STIM1: stromal interaction molecule 1. Scale bars are 10 μm. Images are false colored: CFP, cyan; YFP, green; mRFP, red. All insets show zoomed regions (4x) of structures in dotted rectangles. All experiments were repeated at least 3 times. (PDF 9198 kb) [file 12896_2016_320_MOESM1_ESM.pdf]

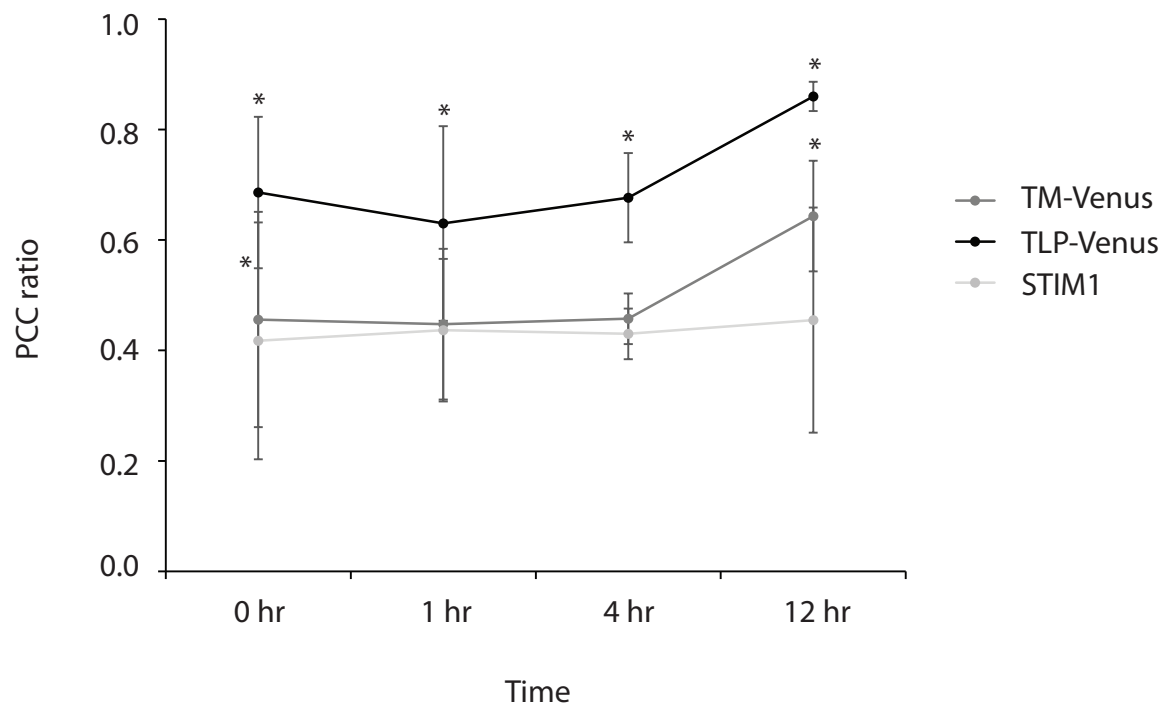

Supplement: Additional file 2: Figure S2. — Line graph showing changes in distribution of plasma membrane labelled Lyn-Ceru compared with ER-labelled STIM1-mRFP, TM-Venus and TLP-Venus in CHO cells, post cyclohexamide treatment. CHO cells transfected with the plasma membrane labelled Lyn-Ceru and the ER labelled STIM1-mRFP, TM-Venus or TLP-Venus. Cells were imaged initially and after 1, 4 and 12 h after incubation with 10 μg/mL cyclohexamide. Y-axis shows PCC of the STIM1-mRFP, TM-Venus or TLP-Venus with Lyn-Ceru. TM: transmembrane domain TLR4, TLP: fusion of TM with signal peptidase cleavage site from human immunoglobulin K, V: Venus fluorescent protein, LC: Lyn-Ceru, STIM1: stromal interaction molecule 1. Error bars indicate s.d. Star indicates significance of p < 0.05. (PDF 238 kb) [file 12896_2016_320_MOESM2_ESM.pdf]
